# The Synergistic Neuroprotective Effect of Honokiol and Magnolol Against Amyloid-β and MPP^+^-Induced Neurotoxicity in SH-SY5Y Cells: An Antioxidant, Molecular Orbital, and ADMET Study

**DOI:** 10.3390/ijms27146096

**Published:** 2026-07-08

**Authors:** Benjamas Suwansukho, Kamonchanok Poempul, Weerasak Samee, Sarin Tadtong

**Affiliations:** 1 Department of Pharmacognosy, Faculty of Pharmacy, Srinakharinwirot University, Ongkharak, Nakhon Nayok 26120, Thailand; benjamassu@g.swu.ac.th (B.S.); khing2772@gmail.com (K.P.); 2 Department of Pharmaceutical Chemistry, Faculty of Pharmacy, Srinakharinwirot University, Ongkharak, Nakhon Nayok 26120, Thailand; weerasak@g.swu.ac.th

**Keywords:** honokiol, magnolol, neuroprotective effect, synergistic effect, amyloid-beta, MPP^+^

## Abstract

Alzheimer’s disease (AD) and Parkinson’s disease (PD) are the two main neurodegenerative diseases and cause disability and death in patients worldwide. Neurodegeneration is characterized by a progressive loss of neuronal function and structure, causing enormous impairment in cognitive–motor function. Magnolol and honokiol are isomeric biphenyl neolignans and have exhibited neuroprotective activity in previous studies. Hence, we assessed and compared honokiol, magnolol, and mixtures of honokiol and magnolol in honokiol/magnolol molar ratios of 1:3, 1:1, and 3:1 in terms of their neurotoxicity, using the cell counting kit-8 (CCK-8) assay, and of their neuroprotective effect on intracellular reactive oxygen species (iROS) against amyloid-beta (Aβ)- and 1-methyl-4-phenylpyridinium ion (MPP^+^)-induced neurotoxicity in SH-SY5Y cells, using the 2′,7′-dichlorodihydrofluorescein diacetate (H_2_DCF-DA) assay. The results showed that honokiol (H) and magnolol (M) at 0.1 μM and the mixtures of honokiol and magnolol in H/M ratios of 1:3, 1:1, and 3:1 at 0.0001 μM exhibited a significant neuroprotective effect of reducing iROS in SH-SY5Y cells where neurotoxicity was induced by Aβ- and MPP^+^ (*p*-value with respect to Aβ-treated cells < 0.005 and *p*-value with respect to MPP^+^-treated cells < 0.0001). Moreover, magnolol and honokiol possess antioxidant properties according to computational molecular analysis with Highest Occupied Molecular Orbital (HOMO)- Lowest Unoccupied Molecular Orbital (LUMO) prediction, 2,2′-azino-bis(3-ethylbenzothiazoline-6-sulfonic acid) (ABTS), 2,2-diphenyl-1-picrylhydrazyl (DPPH), and Ferric Reducing Antioxidant Power (FRAP) assays. The mixtures of honokiol and magnolol exerted synergistic neuroprotective ability at all ratios while showing better antioxidation ability than that of pure magnolol alone but comparable to that of pure honokiol alone. Drug-likeness, Absorption, Distribution, Metabolism, Excretion, and Toxicity (ADMET) prediction, and toxicity profiles showed that both compounds are promising neuroprotective agents and that one of the possible targeting mechanisms is the ROS-mediated oxidative stress pathway. Additional neuronal cell lines and in vivo models are required to determine similar effects or other protective mechanisms involving the neuroprotective ability of honokiol and magnolol.

## 1. Introduction

**Figure 1:**
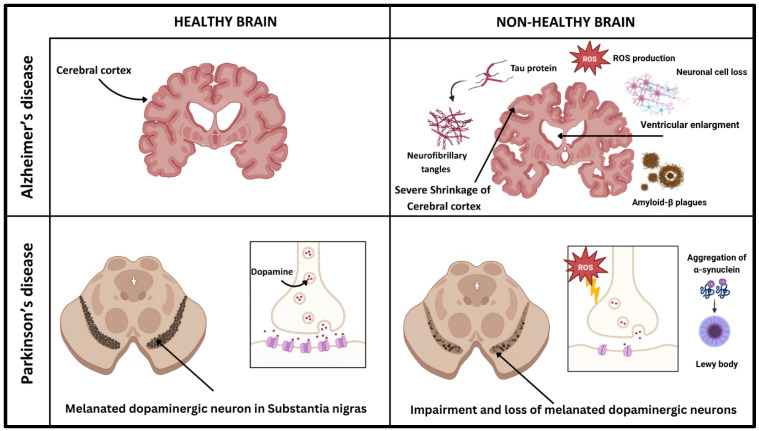
Comparison of histopathological features of Alzheimer’s disease and Parkinson’s disease [13]. Created with BioRender.com.

**Figure 2:**
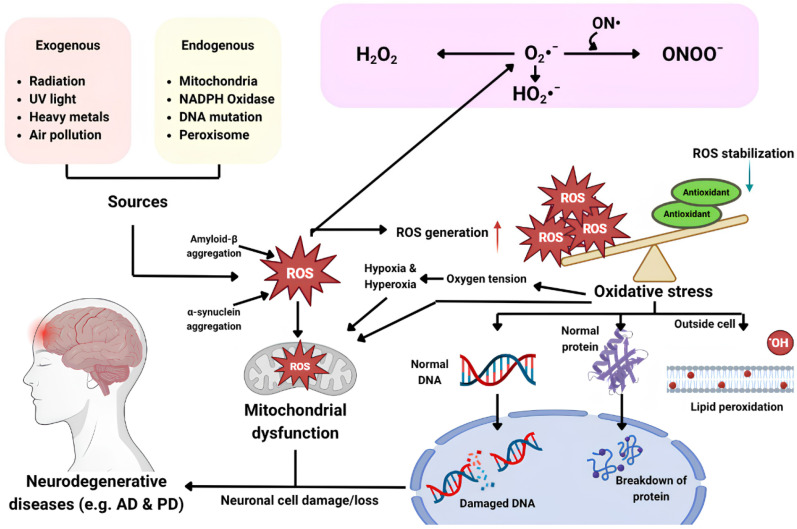
The complex cellular and molecular pathways associated with oxidative stress in AD and PD. The neurotoxins or any stimuli cause reactive oxygen species (ROS), leading to the imbalance between ROS generation (ROS accumulation: a red arrow) and ROS stabilization (antioxidant mechanism dysregulation: a green arrow), followed by oxidative stress occurring in the brain, resulting in the critical role in the pathogenesis of neurodegeneration and neurodegenerative diseases such as AD and PD [16]. Created with BioRender.com.

Globally, many countries are transitioning into aging societies because of demographic transition and the shift to lower fertility and mortality. Epidemiological evidence reported in 2019 showed that more than 60 million individuals worldwide were living with Alzheimer’s disease (AD) and Parkinson’s disease (PD) [1,2]. These neurodegenerative conditions refer to the progressive loss of neuronal structure and function, leading to impaired neural networks and clinical disorders of the brain, impairing activities of daily living, including mobility, communication, cognition, and memory; both genetic and non-genetic factors can be risk factors for both diseases [3,4,5,6]. Despite their distinct clinical neuropathological hallmarks, such as extracellular amyloid-β (Aβ) in AD and the loss of dopaminergic neurons in the substantia nigra par compacta in PD, as shown in Figure 1, accumulating evidence shows that both diseases share several common causes of cell death, including oxidative stress, mitochondrial dysfunction, deoxyribonucleic acid (DNA) mutation, neuroinflammation, and cell apoptosis [7,8]. Excessive production of reactive oxygen species (ROS) plays a central role in disrupting cellular redox homeostasis, DNA fragmentation, and lipid peroxidation; damages neurons; and ultimately leads to progressive neuronal cell loss, as shown in Figure 2 [9]. Thus, antioxidant mechanism-based neuroprotective strategies have attracted considerable interest as potential approaches to preventing neurodegeneration and excessive production of ROS, which can be observed in both Aβ-mediated and MPP^+^-mediated neurotoxicity [10,11]. However, available medications for AD and PD mainly provide symptomatic relief and do not cease disease progression, and there are currently limited therapeutic options targeting these mechanisms [12,13,14], emphasizing the need for neuroprotective agents that target ROS production, as oxidative stress is recognized as a mediator of neuronal injury in both AD and PD [15].

**Figure 3:**
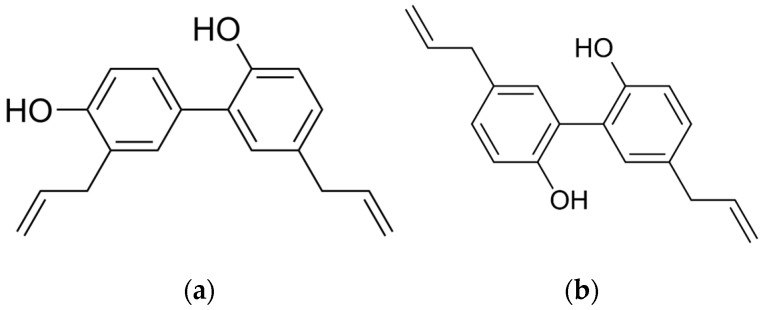
Two-dimensional structures of honokiol and magnolol: (**a**) honokiol; (**b**) magnolol.

Interest in traditional medicines from various cultures has flourished, and people are interested in a longevity lifestyle using a holistic system for planetary health and well-being [17]. The bark of *Magnolia officinalis*, a medicinal plant that is widely used in traditional East Asian medicine, contains two major bioactive isomeric neolignans, honokiol (3,5′-diallyl-4,2′-dihydroxybiphenyl (Figure 3a)) and magnolol (5,5′-diallyl-2,2′-dihydroxybiphenyl (Figure 3b), and both compounds exhibit potent antioxidants, anti-inflammatory, and antiapoptotic properties [18,19]. Previous studies have demonstrated that honokiol and magnolol can reduce ROS generation. Both compounds attenuated MPP^+^- or 1-methyl-4-phenyl-1,2,3,6-tetrahydropyridine (MPTP)-induced neurotoxicity through the modulation of oxidative stress and survival signaling pathways in PD experimental models [10,20]. Similarly, in AD-related models, honokiol (H) and magnolol (M) have been reported to reduce Aβ-induced oxidative damage and neuronal apoptosis while improving pathological and behavioral outcomes in transgenic animal models [21]. This information shows that magnolol and honokiol may act as promising neuroprotective agents targeting ROS-mediated oxidative stress-related pathways.

Despite their structural similarity and overlapping pharmacological activities, honokiol and magnolol exhibit distinct physicochemical properties and molecular signaling profiles, which may influence their neuroprotective efficacy. Furthermore, these compounds naturally coexist in *Magnolia officinalis* and possess complementary biological activities [18], so combined administration may produce additive or synergistic effects [22]. Synergism of phytochemicals is increasingly recognized as an important strategy for enhancing therapeutic efficacy while minimizing the dose [18]. However, most studies independently investigated honokiol and magnolol, making direct comparisons difficult because of differences in experimental models, toxin concentrations, treatment conditions, and outcome measures. Currently, there is very scarce research comparing the neuroprotective efficacy of honokiol and magnolol side by side under identical experimental conditions, as well as evaluating the potential synergistic effects of different honokiol–magnolol combinations against both Aβ- and MPP^+^-induced neuronal injury.

Accordingly, our study aims to both investigate and compare the neuroprotective effects of honokiol (H) and magnolol (M) individually and in the combination molar ratios of 1:3, 1:1, and 3:1 honokiol/magnolol (H:M) using SH-SY5Y cells exposed to Aβ_42_ and MPP^+^ as in vitro models of AD- and PD-related neurotoxicity [23,24,25], respectively. The emphasis of the modulation of intracellular oxidative stress contributes to their neuroprotective actions. In addition, the direct antioxidant capacities of honokiol, magnolol and the mixtures were determined using DPPH, ABTS, and FRAP assays, while frontier molecular orbital (FMO) analysis was used to examine their theoretical antioxidant potential. To support the assessment of their drug development prospects, in silico analyses on drug-likeness, pharmacokinetic properties, ADMET characteristics and toxicity prediction profiles were also performed. By integrating computational, chemical, and biological approaches, this study aims to clarify the relative contribution of honokiol and magnolol and determine whether their combination offers advantages and shows synergistic effects over individual compound strategies for oxidative stress-mediated neurodegeneration.

## 2. Results

### 2.1. Antioxidant Activities of Honokiol and Magnolol

**Figure 4:**
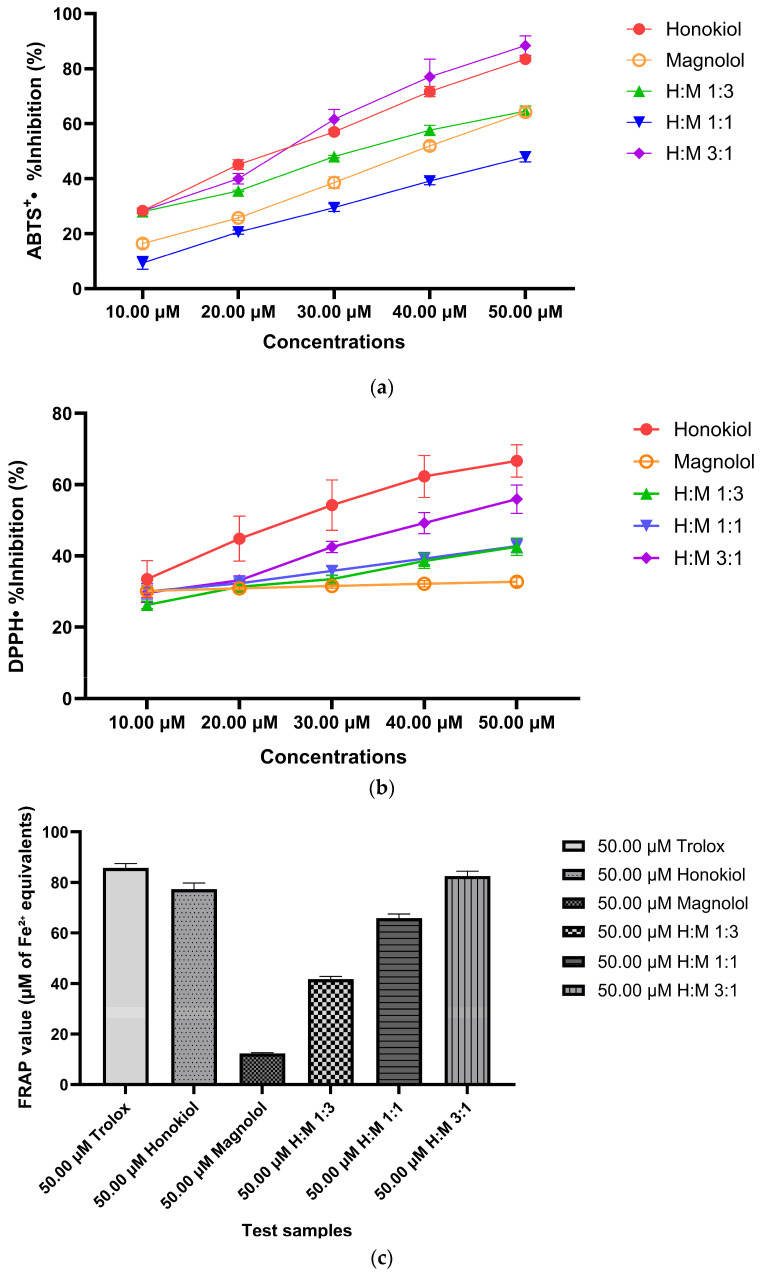
Antioxidant activities of honokiol and magnolol. (**a**) ABTS radical scavenging activity, (**b**) DPPH radical scavenging activity of honokiol, magnolol and all ratios (1:3, 1:1, and 3:1 H:M) of the mixtures between honokiol and magnolol at 10.00–50.00 μM, and (**c**) FRAP assay results with honokiol, magnolol and H/M 1:3, 1:1, and 3:1 at 50.00 μM, while Trolox was used as a known positive control at 50.00 µM (triplicate, *n* = 3). The abbreviation H/M means the mixtures of honokiol and magnolol.

As the results show in Figure 4a–c, we tested honokiol, magnolol and the mixtures of honokiol and magnolol in all three ratios (1:3, 1:1, and 3:1) at 10.00–50.00 μM. IC_50s_ of honokiol, magnolol, the mixtures of honokiol and magnolol at 1:3, 1:1, 3:1 on ABTS radical scavenging assays are 24.80 ± 0.64 µM, 38.76 ± 0.33 µM, 33.42 ± 0.70 µM, >50 µM, and 17.26 ± 0.52 µM, respectively; the IC_50_ of honokiol, magnolol, H/M in all three ratios of 1:3, 1:1, 3:1 on DPPH radical scavenging assays are 27.19 ± 5.70 µM, >50 µM, >50 µM, >50 µM, and 41.75 ± 3.36 µM, respectively; and the FRAP values of honokiol, magnolol, and H/M 1:3, 1:1, 3:1 at 50.0 µM on FRAP assays are 77.05 ± 2.04 µM, 12.29 ± 0.25 µM, 41.61 ± 0.85 µM, 65.73 ± 1.32 µM, 82.38 ± 1.55 µM of Fe^2+^ equivalent, respectively. It shows that honokiol, magnolol and H/M in all three ratios have concentration-dependent inhibition.

### 2.2. Computer Molecular Analysis for Honokiol and Magnolol

#### 2.2.1. Molecular Orbital Energy Analysis for Honokiol and Magnolol

**Figure 5:**
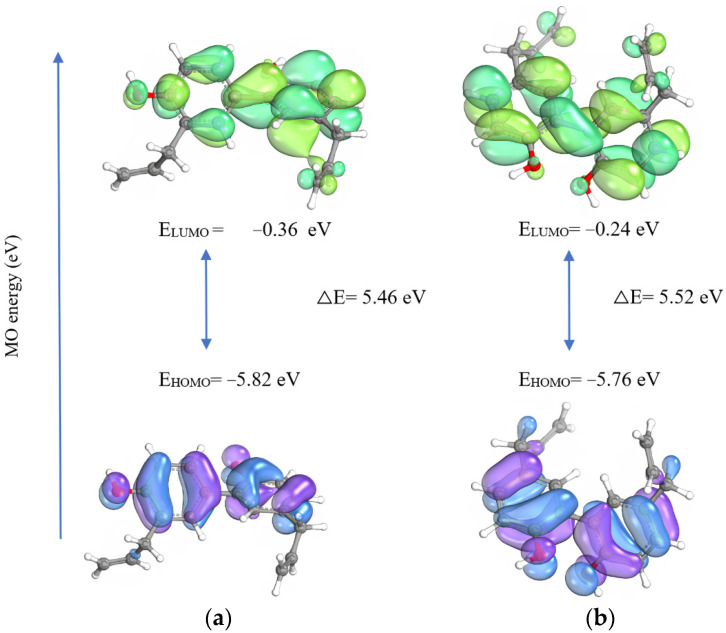
Diagram showing HOMO-LUMO energy gap (eV) calculated as HOMO and LUMO energy shift in (**a**) honokiol and (**b**) magnolol and their optimized geometry at the B3LYP/def2-SVP level of theory. The green clouds over structures of honokiol and magnolol are the energy cloud in the LUMO level, and the blue-purple clouds over structures of honokiol and magnolol are the energy cloud in the HOMO level, visualized by IboView (v20211019).

The experiment was performed according to the method described by Tantinithiphong et al., 2025 [26]. The highest occupied molecular orbitals (HOMOs) and lowest unoccupied molecular orbitals (LUMOs) of the antioxidant play a major role in the chemical reactivity of antioxidants. These are very significant in the study of charge transfer between antioxidants and ROS. A higher HOMO energy reflects enhanced electron-donating capacity, and a lower LUMO energy reflects enhanced electron-accepting capacity. As the result shows in Table 1, the HOMO-LUMO energy gaps of honokiol and magnolol are ΔE = 5.46 eV and ΔE = 5.52 eV, respectively, as shown in Figure 5. The lower HOMO-LUMO energy gap (ΔE) indicates better antioxidant activity.

#### 2.2.2. Drug-Likeness and ADMET Analysis for Honokiol and Magnolol

**Figure 6:**
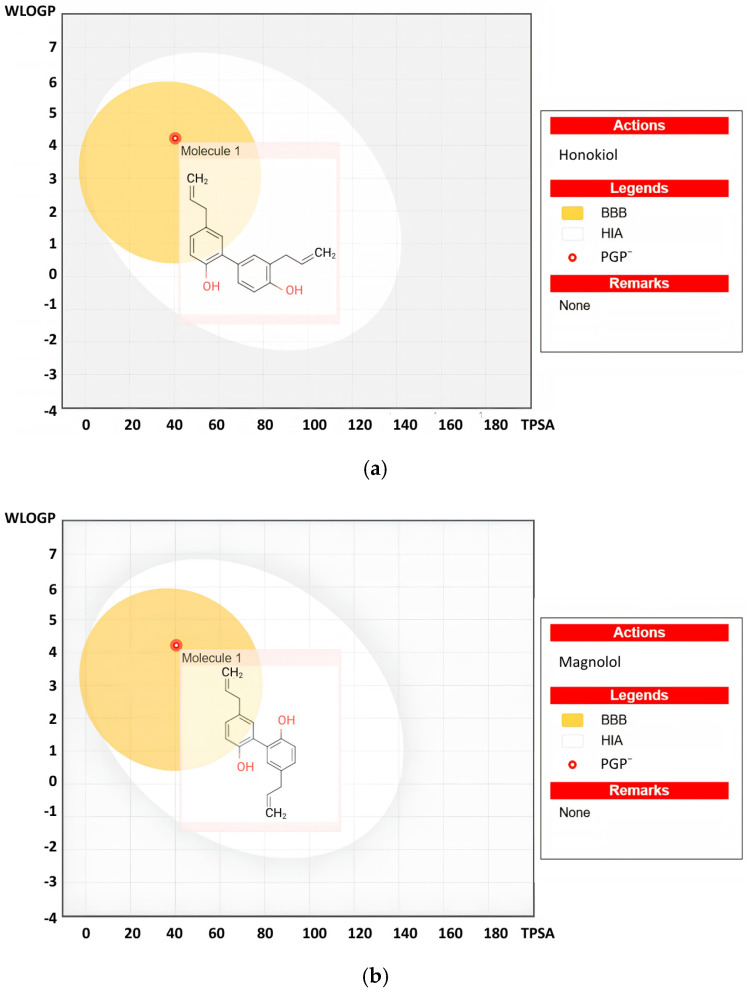
BOILED-EGG model of (**a**) honokiol and (**b**) magnolol, computed by SwissADME. Abbreviations: BBB—Blood–Brain Barrier, HIA—Human Gastrointestinal Absorption, PGP^−^—non-substrate of the P-glycoprotein.

For the physicochemical properties analysis of honokiol and magnolol structures with SwissADME, these subtle structural variations significantly influence pharmacokinetics, membrane interaction, and biological activity, as the result shows in Table 2. The physicochemical properties of honokiol and magnolol show that both structures have high lipophilicity and moderate molecular weight (<500 Da) with low polarity, and balanced lipophilicity shows that high gastrointestinal (GI) absorption is more likely to cross the blood–brain barrier (BBB), representing the likelihood of sufficient oral bioavailability, according to the BOILED-EGG model in Figure 6. SwissADME analysis also suggests that neither compound is a substrate of P-glycoprotein (P-gp), indicating that efflux transport mechanisms are unlikely to significantly limit their bioavailability. However, both compounds show potential interactions with cytochrome P450 enzymes, which may influence metabolic stability and drug–drug interaction potential, as shown in Table 3.

#### 2.2.3. In Silico Organ Toxicity and Drug Metabolism via the Cytochrome P450 System Prediction on ProTox 3.0 of Honokiol and Magnolol

We conducted in silico toxicity analysis of honokiol and magnolol with ProTox 3.0. These subtle structural variations influence and cause different probabilities for organ toxicity, as the result shows in Table 4. The relatively high probabilities for cardiotoxicity inactivity suggest a low likelihood of adverse cardiac effects, while slightly lower probabilities are observed for neurotoxicity and nephrotoxicity. Nonetheless, the overall pattern suggests that both honokiol and magnolol possess a low predicted risk of organ-specific toxicity.

The drug metabolism predictions via the cytochrome P450 (CYP450), computed by ProTox3.0, are shown in Table 5. The prediction suggests that honokiol and magnolol may cause metabolic involvement with CYP2C19 and CYP2C9, and other major isoforms, including CYP1A2, CYP2D6, CYP3A4, and CYP2E1, were shown to be inactive. It suggests that honokiol and magnolol have potential interactions with the CYP2C subfamily; however, this only reflects potential metabolic involvement with CYP450, not showing a drug–drug interaction risk.

### 2.3. Neuronal Viabilities of Honokiol and Magnolol Against Untreated SH-SY5Y Cells

**Figure 7:**
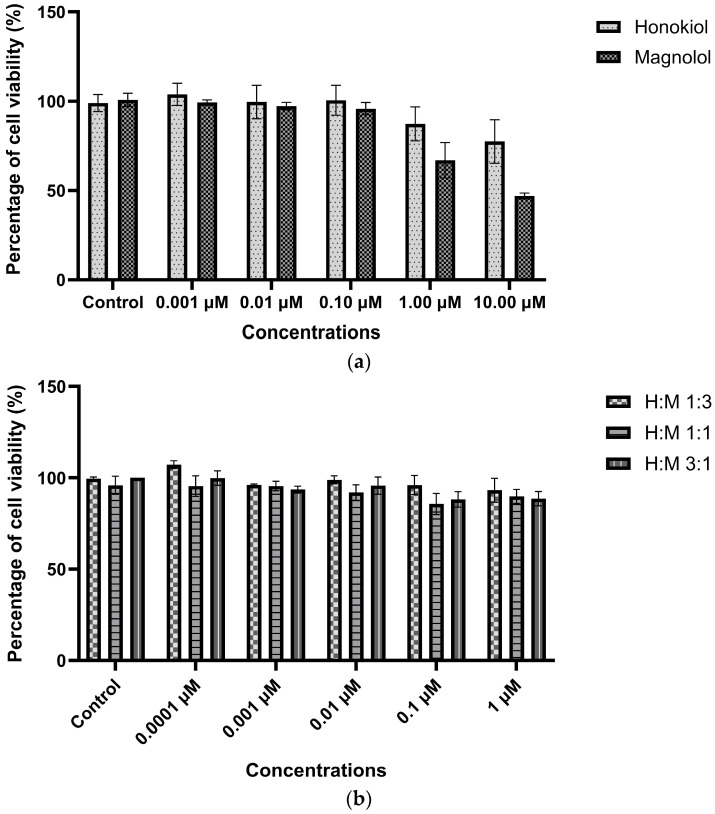
(**a**) Cell viability assay on SH-SY5Y cells treated with honokiol and magnolol at various concentrations (0–10.00 µM) for 24 h. (**b**) Cell viability assay on SH-SY5Y cells treated with H/M 1:3, 1:1, 3:1 at various concentrations (0–1.00 µM) for 24 h. The data are expressed as the mean ± standard deviation (SD) of %cell viability (triplicate, *n* = 3).

Figure 7a presents the effects of different concentrations of honokiol and magnolol (0–10.00 µM) on SH-SY5Y cells. In the cytotoxicity assay, the result indicated that the %cell viability of honokiol at 0.001, 0.01, and 0.1 μM showed ranges of 103.80 ± 6.20%, 99.51 ± 9.37%, 100.51 ± 8.35%, respectively, and showed no statistical difference among them and the control (*p*-value > 0.05). A similar effect was found in magnolol at 0.001, 0.01, and 0.1 μM, which showed %cell viability ranges of 99.29 ± 1.47%, 97.26 ± 2.13%, 97.74 ± 0.50%, respectively, and also showed no statistical differences among them and the control too (*p*-value > 0.05). Moreover, there was no statistical difference between %cell viability of magnolol and honokiol at 0.1 μM (*p*-value > 0.05). Hence, we selected the highest concentration which showed no neuronal toxicity (0.1 μM) to test for their neuroprotective ability.

In addition, the result indicated that the mixtures of honokiol and magnolol in three ratios at 1:3, 1:1, and 3:1 showed %cell viability of H/M at 0.0001, 0.001, and 0.01 μM over 90% of cell viability and also showed no statistical differences among them and the control (*p*-value > 0.05). Moreover, there was no statistical difference between the %cell viability of H/M at all three ratios at 0.0001 μM (*p*-value > 0.05). Hence, we selected the lowest concentration (0.0001 μM) of all three ratios as %cell viabilities of 107.19 ± 2.16%, 95.40 ± 5.60%, and 99.79 ± 3.98%, respectively, which reflects no neuronal toxicity for their neuroprotective ability and synergistic effect of the mixtures of honokiol and magnolol, as shown in Figure 7b.

### 2.4. Neuroprotective Effects of Honokiol and Magnolol Against Aβ-Induced and MPP^+^-Induced SH-SY5Y Cells

**Figure 8:**
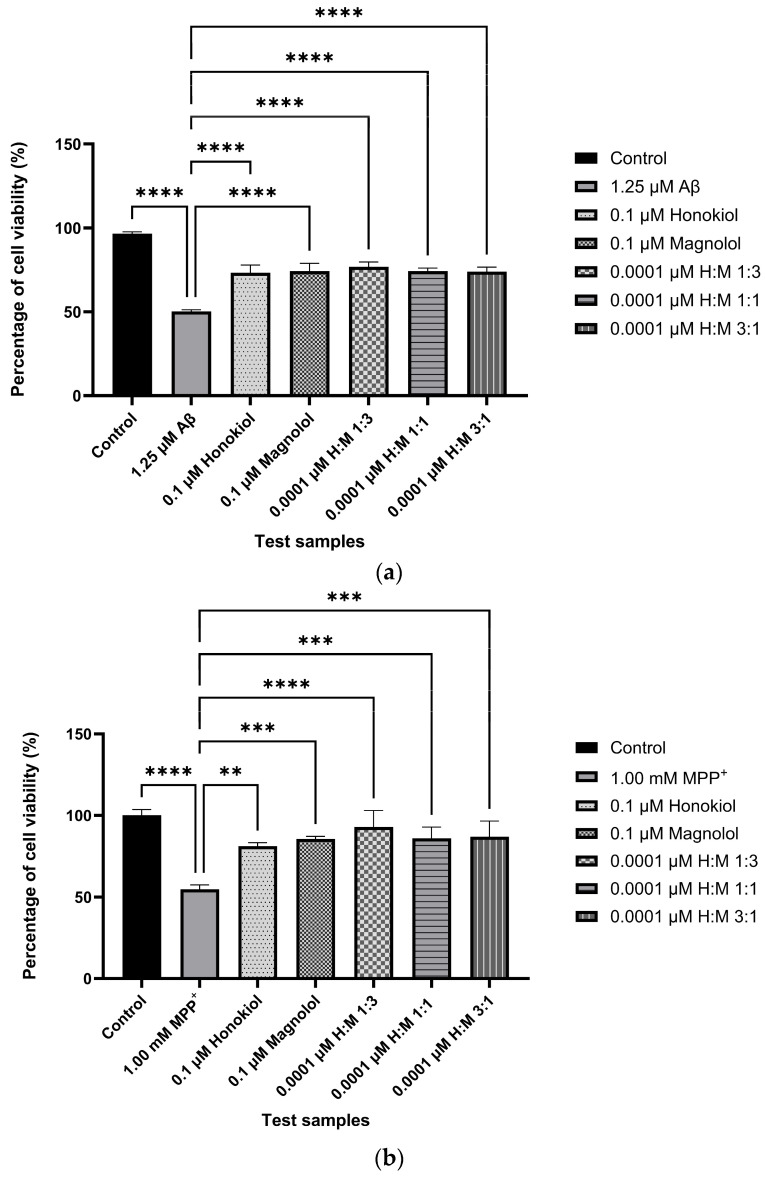
Cell viability of SH-SY5Y cells treated with various substances: (**a**) %cell viability of Aβ-induced SH-SY5Y cells and treated with honokiol and magnolol at 0.1 μM and H/M in all three ratios at 0.0001 μM; (**b**) %cell viability of MPP^+^-induced SH-SY5Y cells and treated with honokiol and magnolol at 0.1 μM and H/M in all three ratios at 0.0001 μM. The data are expressed as the mean ± standard deviation (SD) of %cell viability (triplicate, *n* = 3). ** indicates *p*-value < 0.005, *** indicates *p*-value < 0.0005, **** indicates *p*-value < 0.0001 (compared with neurotoxin-treated group).

Neuroprotective assay of the test samples showed that the SH-SY5Y cells pretreated with 0.1 μM of honokiol or magnolol individually and 0.0001 μM of the mixtures of honokiol and magnolol in three ratios as 1:3, 1:1, and 3:1 for 1 h, followed by 1.25 μM of Aβ_42_-induced neurotoxicity for 24 h, express the average percentages of cell viability for honokiol and magnolol individually at 0.1 μM as 73.35 ± 4.56% and 74.30 ± 4.60%, respectively, while 0.0001 μM of H/M in three ratios of 1:3, 1:1, and 3:1 expresses the average percentages of cell viability as 76.80 ± 2.84%, 74.23 ± 1.85%, and 73.98 ± 2.66%, respectively. The Aβ_42_-treated group possessed the average percentages of cell viability at 50.27 ± 0.93%. Figure 8a shows that honokiol, magnolol and H/M in all three ratios of 1:3, 1:1, and 3:1 significantly increased the cell viability of the Aβ_42_-treated cells about 46%, 48%, 52%, 48%, and 47%, respectively, and no statistical difference was observed between all test samples in the pretreated group (*p*-value > 0.05).

Furthermore, the average percentages of cell viability for pretreated honokiol and magnolol individually at 0.1 μM followed by 1.00 mM (1000 μM) of MPP^+^-induced neurotoxicity SH-SY5Y cells were 81.03 ± 2.14% and 85.54 ± 1.59%, respectively, while 0.0001 μM of H/M in all three ratios of 1:3, 1:1, and 3:1 was 92.95 ± 9.99%, 85.91 ± 6.90%, 86.89 ± 9.60%, and the MPP^+^-treated group showed average percentages of cell viability at 54.63 ± 2.63% (Figure 8b). Honokiol, magnolol, and H/M in all three ratios significantly increased the cell viability of the MPP^+^-treated cells about 48%, 57%, 70%, 57%, and 59%, respectively, and there was no statistical difference observed between all test samples in the pretreated group (*p*-value > 0.05).

### 2.5. Neuroprotective Activity of Honokiol and Magnolol on Intracellular Reactive Oxygen Species (iROS) with Fluorescence Probe H_2_DCF-DA Assay

**Figure 9:**
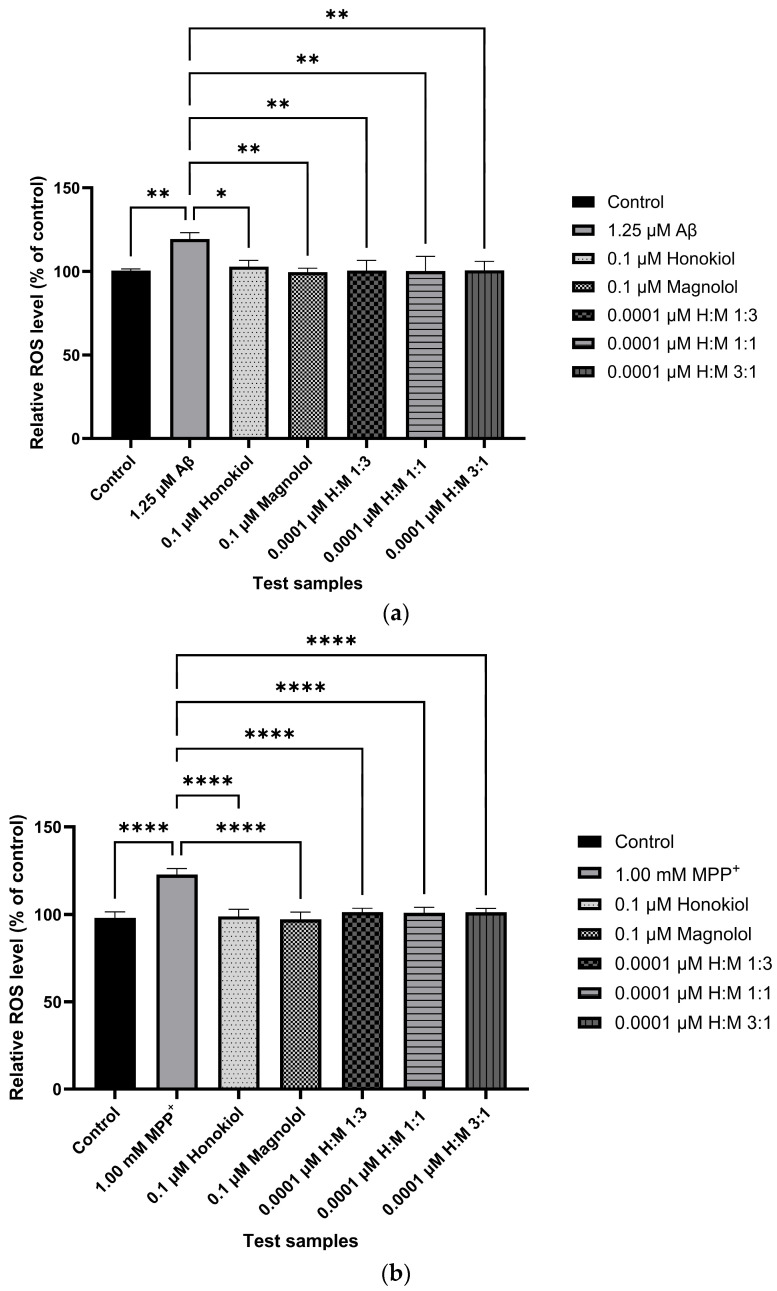
The inhibition of iROS of honokiol and magnolol is shown as fluorescence density of the cell probed with H_2_DCF-DA assay, expressing result as %ROS level compared with untreated cells: (**a**) %ROS level of Aβ_42_-induced SH-SY5Y cells and treated with honokiol and magnolol at 0.1 μM, and H/M in all three ratios at 0.0001 μM, (**b**) %ROS level of MPP^+^-induced SH-SY5Y cells and treated with honokiol and magnolol at 0.1 μM and H/M in all three ratios at 0.0001 μM. The data are expressed as the mean ± standard deviation (SD) of %iROS (triplicate, *n* = 3) (* indicates *p*-value < 0.05, ** indicates *p*-value < 0.005, **** indicates *p*-value < 0.0001 compared with neurotoxin-treated group). Abbreviation: H/M—the mixtures of honokiol and magnolol.

According to Figure 9a, honokiol and magnolol at 0.1 μM and H/M 1:3, 1:1, and 3:1 at 0.0001 μM possessed neuroprotective effects via antioxidant activity by reducing intracellular reactive oxygen species (iROS) and showed no statistical difference among all test samples (*p*-value > 0.05). Thus, the pretreated neuronal cells were protected from intracellular ROS induced by Aβ_42_ compared to untreated Aβ_42_-induced SH-SY5Y. The %iROS of untreated SH-SY5Y cells was 119.25 ± 0.63%, while the inhibition of iROS of 0.1 μM honokiol, 0.1 μM magnolol, H/M 1:3, 1:1, and 3:1 at 0.0001 μM exhibited the %iROS fluorescence density of the cell probed with the H_2_DCF-DA assay at 102.67 ± 0.47%, 99.47 ± 0.72%, 100.53 ± 1.55%, 100.00 ± 1.03%, and 100.53 ± 0.68%, respectively (* *p*-value < 0.05, ** *p*-value < 0.005 versus Aβ_42_-induced). The results reflected that honokiol, magnolol, and all three ratios of the mixtures of honokiol and magnolol helped reduce the iROS induced by Aβ_42_ at about 14%, 17%, and 16% for all three ratios, respectively.

Similarly, Figure 9b shows that the pretreated neuronal cells with honokiol and magnolol at 0.1 μM and M:H 1:3, 1:1, and 3:1 at 0.0001 μM were significantly protected from intracellular ROS induced by MPP^+^ compared to untreated MPP^+^-induced SH-SY5Y cells (*p*-value > 0.05). The inhibition of iROS by honokiol and magnolol at 0.1 μM and H/M 1:3, 1:1, and 3:1 at 0.0001 μM expressed the %iROS fluorescence density of the cell probed with the H_2_DCF-DA assay, which was 102.72 ± 1.97%, 96.60 ± 1.75%, 98.64 ± 1.03%, 97.28 ± 1.59%, and 101.36 ± 1.57% respectively, while the %iROS of untreated MPP^+^-induced SH-SY5Y cells was 122.45 ± 1.41% (* *p*-value < 0.05 versus MPP^+^-induced). Honokiol, magnolol and H/M in all three ratios of 1:3, 1:1, and 3:1 helped reduce the iROS induced by MPP^+^ by about 16%, 21%, 19%, 20%, and 17%, respectively, and showed no statistical difference between the honokiol- and magnolol-pretreated groups (*p*-value > 0.05). These results reflected the synergistic neuroprotective effect of honokiol and magnolol by reducing the dose being used when compared to that of single pure honokiol and magnolol.

## 3. Discussion

Frontier molecular orbital (FMO) analysis with density functional theory (DFT) calculation showed a slight difference between honokiol and magnolol. The HOMO energy reflects electron-donating ability, while the HOMO-LUMO energy gap (ΔE) indicates molecular reactivity and is a good indicator for describing the potential of a molecule in terms of radical scavenging. Honokiol exhibited slightly lower HOMO energy (−5.82 eV) and HOMO-LUMO energy gap (5.46 eV) than magnolol (−5.76 eV and 5.52 eV, respectively), as the lower energy gap between HOMO and LUMO energy reflects a higher electron-donating ability. Moreover, honokiol showed slightly lower chemical hardness (η = 2.73) and higher electronegativity (χ = 3.09) compared with magnolol (η = 2.76, χ = 3.00). These results suggest that honokiol possesses slightly higher chemical reactivity and slightly greater electron-transfer capability than magnolol and shows better antioxidant activity than magnolol. ABTS, DPPH and FRAP assays revealed that honokiol demonstrated stronger radical scavenging ability (according to ABTS and DPPH assays) and reducing power (according to FRAP assay) than magnolol at all tested concentrations (10.0–50.0 μM). The ABTS, DPPH, and FRAP assays also showed that among all the tested mixtures between honokiol and magnolol, H/M in a 3:1 ratio mixture at 50.0 μM produced the highest ABTS inhibition and FRAP value, approximately 88% and 82 µM of Fe^2+^ equivalents, respectively, compared with 83% and 77 µM of Fe^2+^ equivalents for honokiol and 63% and 12.3 µM of Fe^2+^ equivalents for magnolol. However, DPPH inhibition at 50.0 μM of the H/M 3:1 ratio (56%) was lower than 50.0 μM honokiol alone (66%), while magnolol alone showed the weakness of overall antioxidation activities. Interestingly, H/M at 1:1 and 1:3 ratios was less active than honokiol but more active than magnolol across the evaluated antioxidant assays. These outcomes suggest that the antioxidant activity of honokiol and magnolol is ratio-dependent, and the mixture of honokiol and magnolol possessed better antioxidation ability than pure magnolol. The superior performance of the 3:1 mixture arises from complementary radical scavenging mechanisms as the higher HOMO energy of honokiol indicates a greater tendency to donate electrons during SET-mediated antioxidant reactions, suggesting that honokiol may initiate radical quenching more rather than magnolol. Computational analysis shows slightly different HOMO-LUMO energy gaps of honokiol and magnolol but a distinct chemical reaction between honokiol, magnolol, and H/M in all three ratios of 1:3, 1:1, 3:1 due to various reasons, such as the fact that the structure of magnolol is more symmetric, causing stronger crystal packing, leading to lower solubility and reducing apparent antioxidant effects compared to honokiol [27]. The results also indicate that excessive magnolol proportions may dilute the contribution of honokiol, explaining the lower activity observed in the H/M 1:1 and 1:3 formulations. According to a previous study, Single Electron Transfer (SET) [28] can explain the effects of magnolol and honokiol on ABTS scavenging activity. Honokiol was consistent with its smaller HOMO-LUMO gap, which enhanced electron-donating capacity, leading to better antioxidative activity. Furthermore, HOMO electron density was found distributing over the phenolic hydroxyl groups, emphasizing their direct contribution to antioxidant activity through electron donation and radical stabilization. Compounds exhibiting lower ionization potential generally demonstrated greater SET efficiency because less energy was required to remove an electron [29].

SwissADME analysis indicated that both honokiol and magnolol satisfy Lipinski’s rule of five, exhibiting low molecular weight (266.3 g/mol), moderate lipophilicity (logP = 4.22), low TPSA (40.46 Å^2^), and favorable hydrogen bonding [30]. Importantly, both compounds were predicted to possess high gastrointestinal absorption and blood–brain barrier (BBB) permeability as BBB penetration is a critical requirement for therapeutic agents targeting neurodegenerative diseases, such as Alzheimer’s and Parkinson’s. The predicted BBB permeability observed in the present study supports previous reports, demonstrating that honokiol and magnolol readily reach brain tissue and exert neuroprotective effects through the modulation of oxidative stress, mitochondrial dysfunction and apoptosis pathways [31], and the BBB permeability assay is required to evaluate and confirm the BBB permeability prediction. Furthermore, both compounds display an identical oral bioavailability score, which is 0.55, suggesting adequate oral absorption [32]. These properties strengthen their potential as lead compounds for the development of neural protective agents against oxidative stress-mediated neuronal injury, while the toxicity profiles on ProTox 3.0 demonstrated that honokiol and magnolol were inactive toward hepatotoxicity, neurotoxicity, nephrotoxicity, respiratory toxicity and cardiotoxicity. The probability values indicated a general favorable safety profile for both compounds. Interestingly, honokiol has a slightly higher probability for cardiotoxicity activities as 0.84 and neurotoxicity activities as 0.53 compared with magnolol (0.82 and 0.50, respectively), suggesting marginally better predicted safety. Previous toxicological assessments of magnolia-derived compounds showed similarly reported low toxicity and acceptable safety margins, supporting their suitability for a long-term therapeutic application [33], consistent with the present study, which showed that honokiol, magnolol and H/M in all three ratios are nontoxic against SH-SY5Y cells. The cytochrome P450 predictions indicated that both compounds may interact with CYP2C19 and CYP2C9, while showing drug–drug interactions on CYP1A2, CYP2D6, CYP3A4 and CYP2E1 metabolism [34,35]. These results suggest that honokiol and magnolol possessed favorable pharmacokinetic characteristics; however, potential herb–drug interactions involving CYP2C19 and CYP2C9 should be considered during future clinical development. Using the mixture of honokiol and magnolol at lower concentrations than individually pure compounds may help reduce the toxicity of these two molecules.

The accumulation of Aβ peptide and induction of MPP^+^ are known to stimulate excessive ROS production, mitochondrial dysfunction, lipid peroxidation and activation of apoptosis pathways in neuronal cells [36,37]. Aβ peptide is a derivative product of amyloid precursor protein (APP) through sequential cleavages, first by beta-site APP cleaving enzyme 1 (BACE1) and then by γ-secretase complex, and the common isoforms are Aβ_1-40_ and Aβ_1-42_ [38]. In AD, Aβ_1-42_ aggregates and forms Aβ plagues or directly binds with the abnormal increase in zinc. Copper and iron lead to metal-Aβ accumulation in the brain. Aβ accumulation leads to ROS generation and ATP depletion, which causes mitochondrial dysfunction, and inflammation, which causes oxidative damage in cells [39]. Meanwhile, MPP^+^ (1-methyl-4-phenylpyridinium) is the highly toxic active metabolite of MPTP (1-methyl-4-phenyl-1,2,3,6-tetrahydropyridine) and most widely used neurotoxin for PD models [38,40]. The mechanism of MPP^+^ toxicity is attributed to a series of interconnected cellular events. Extracellular MPP^+^ is selectively internalized into dopaminergic neurons through the dopamine transporter (DAT), after which it accumulates within mitochondria owing to its high affinity for the mitochondrial membrane potential. Mitochondrial MPP^+^ subsequently inhibits complex I of the electron transport chain, leading to impaired oxidative phosphorylation, ATP depletion, and excessive production of ROS. The resulting oxidative stress and mitochondrial dysfunction activate apoptotic signaling pathways, culminating in dopaminergic neuronal cell death [41]. According to previous studies, high concentrations of Aβ (1.00 μM) induced oxidative stress and catalase activity and decreased cell viability [25], and 1.00 mM MPP^+^ decreased 10% cell viability, whereas oxygen consumption was reduced by nearly 70% across major respiratory states. These observations indicate that mitochondrial dysfunction is an early and prominent event in MPP^+^-induced neurotoxicity. The substantial impairment of respiratory activity suggests that MPP^+^ selectively targets the mitochondrial bioenergetic function, possibly through the inhibition of oxidative phosphorylation accompanied by a reduction in mitochondrial mass, ultimately contributing to neuronal degeneration [10]. Our study, treatment with 1.25 μM Aβ_42_ and 1.00 mM MPP^+^, significantly increased intercellular ROS levels to approximately 120% and 122% of control, respectively, confirming the successful induction of oxidative stress. This suggested that both 1.25 μM Aβ_42_ and 1.00 mM MPP^+^ could directly affect mitochondria, according to the previous studies. Our results expressed the ability of honokiol, magnolol and H/M mixtures to suppress induced ROS production (Figure 9). Moreover, Figure 8a,b and Figure 9a,b show the similarity of increasing %cell viability and the iROS reduction in pretreatment with honokiol, magnolol, and H/M mixtures (1:3, 1:1, and 3:1) against Aβ_42_- and MPP^+^-induced SH-SY5Y cells, and it is likely mediated through multiple antioxidant pathways, including direct free-radical scavenging, enhancement of endogenous antioxidant enzymes, such as superoxide dismutase (SOD) and catalase (CAT), preservation of mitochondrial function and activation of the Nrf-2 signaling pathway, as Nrf-2 is a master regulator of cellular antioxidant defense that controls the expression of genes encoding HO-1, SOD and glutathione-related enzymes. Activation of this pathway has been widely recognized as a promising therapeutic strategy for neurodegenerative diseases [42,43].

Many studies showed that a reduction in Aβ accumulation and the protection of cells from MPP^+^ toxicity, as well as the enhancement of SOD and CAT activities, can improve neurodegeneration under oxidative stress conditions. Likewise, honokiol and magnolol have been reported to attenuate oxidative stress, mitochondrial dysfunction and neuroinflammation in several neurological disease models [42,44]. Our study demonstrated that pretreatment with magnolol and honokiol individually and honokiol–magnolol combinations (1:3, 1:1, and 3:1) significantly attenuated intracellular ROS accumulation, with higher cell viability in Aβ_42_- and MPP^+^-induced SH-SY5Y cells compared with the untreated group. Interestingly, magnolol and honokiol individually and all three ratios of honokiol–magnolol combinations (1:3, 1:1, and 3:1) restored iROS levels close to the untreated control, although no significant differences were observed among the pretreated groups. Moreover, the 3:1 honokiol–magnolol mixture ratio is the most promising ratio toward ROS reduction, as H/M in 3:1 exhibited a trend toward a greater ROS reduction. The comparable effect observed with H/M at a ratio of 3:1 may indicate a favorable pharmacological interaction between honokiol and magnolol, potentially allowing effective ROS reduction while reducing the amount of each individual compound required, thus minimizing concentration-related side effects while maintaining antioxidant effects. However, no significant differences were detected. Further concentration–response and combination analyses are necessary, suggesting that all three tested ratios (1:3, 1:1, and 3:1) may not represent the optimal ratio for achieving maximal antioxidant activity. Future investigations with a wider range of honokiol–magnolol ratios are needed to identify the most effective formulation and further characterize to confirm whether a true synergistic interaction exists and/or other mechanisms may be involved, such as anti-inflammatory inhibition of pro-inflammatory mediators (IL-1β, TNF-α and IL-6) [45,46,47]. As these results are limited to the SH-SY5Y cellular models, further studies using additional neuronal cell lines and in vivo models are required to determine whether similar effects are observed under more physiologically relevant conditions.

Our study demonstrated a combined evaluation of the antioxidant and neuroprotective effects of honokiol and magnolol through in silico prediction, quantum chemical analysis, ADMET assessment, and biological antioxidant activity. As for the results, both compounds possess drug-like properties and antioxidant properties. The resemblance between radical scavenging activities and intracellular ROS suppression shows that the electron-donating properties predicted by DFT calculations are biologically relevant. Although the mixtures of honokiol and magnolol at the 3:1 ratio demonstrated the highest chemical antioxidant activity among the three mixture ratios, all test samples effectively attenuated Aβ_42_- and MPP^+^-induced oxidative stress in SH-SY5Y cells, indicating that direct radical scavenging represents one of the initial protective mechanisms that is subsequently enhanced by cellular antioxidant responses, and it supports oxidative stress reduction as one of the mechanisms underlying the neuroprotective effects of honokiol, magnolol, and the mixtures of honokiol and magnolol at all ratios. In addition, the mixtures of honokiol and magnolol, especially at the 3:1 ratio, showed enhanced antioxidant activity and synergistic neuroprotective ability. However, in our experiment, Aβ_42_- and MPP^+^ upregulated the iROS by only 20%, while the neuroprotection ability of honokiol, magnolol, and the M/H mixtures can restore neuronal viability to 46–70%. Hence, we suggest that other mechanisms of action must be involved in this neuroprotective effect. Therefore, future studies should investigate downstream antioxidant pathways, including Nrf-2/HO-1 activation, glutathione homeostasis, mitochondrial membrane potential, lipid peroxidation, BAX/Bcl-2 regulation, caspase-3 activation, and apoptosis signaling, to further elucidate the molecular mechanism underlying the observed neuroprotective effects [48,49].

## 4. Materials and Methods

### 4.1. Materials and Chemicals

Cell Counting Kit-8 (CCK-8) was purchased from Dojindo Laboratories (Nagasaki, Japan). Human Neuroblastoma Cell Line: SH-SY5Y ATCC^®^ CRL-2266^TM^ and Eagle’s Minimum Essential Medium (EMEM) were purchased from American Type Culture Collection (Manassas, VA, USA). Fetal Bovine Serum (FBS) was purchased from Sigma-Aldrich (Barueri, São Paulo, Brazil). F-12 nutrient mixture and antibiotic–antimycotic solution were purchased from Gibco (Carlsbad, CA, USA). H_2_DCF-DA assay was purchased from Invitrogen (Carlsbad, CA, USA). Amyloid-β 42-1 (Aβ_42-1_) reverse human, MPP^+^ iodide and trypsin were purchased from Sigma-Aldrich (St. Louis, MO, USA). Magnolol and honokiol were purchased from My Skin Recipe (Bangkok, Thailand), purity ≥ 99%.

For antioxidant test activities, ABTS diammonium salt [2,2′-Azino-bis (3-ethylbenzothiazoline-6-sulfonic acid diammonium salt)] was purchased from Sigma Co. (Sigma-Aldrich, Hamburg, Germany). Acetic Acid Glacial was purchased from QRëC (Auckland, New Zealand). 2,2-Diphenyl-1-picrylhydrazyl (DPPH) and Trolox were purchased from Aldrich (Hamburg, Germany). Ferric chloride hexahydrate (FeCl_3_·6H_2_O) was purchased from Panreac (Barcelona, Spain). Ferrous sulfate heptahydrate (FeSO_4_·7H_2_O) was purchased from LOBA CHEMIE PVT. LTD (Mumbai, India). Sodium acetate trihydrate (NaOAc·3H_2_O) was purchased from UNIVAR (Ingleburn, NSW, Australia). 2,4,6-Tripyridyl-s-triazine (TPTZ) and ethanol were purchased from Supelco (Buchs, Switzerland). The chemicals were purchased from many sources. All other chemicals were of analytical grade.

### 4.2. Antioxidant Activities Assay

#### 4.2.1. ABTS^+^• Scavenging Assay

According to a previous study, the protocol for ABTS^+^• radical scavenging assay is followed and slightly modified from Wongsukkasem et al., 2018 [50].

The ABTS radical cation reagent was prepared by mixing 7 mM ABTS with 2.45 mM potassium persulfate (1:1, *v*/*v*) overnight. The absorbance of the resulting solution was measured prior to dilution, and ethanol was subsequently added to adjust the absorbance to an appropriate working value (0.80–1.00).

For the antioxidant activity assay, 20 µL of honokiol, magnolol, and the mixture of honokiol (H) and magnolol (M) at molar ratios of H/M 1:3, 1:1, 3:1 solution (at final concentration is 10–50 µM) was added to 180 µL of ABTS reagent in individual wells of a 96-well microplate. After incubation for 6 min, absorbance was recorded at 734 nm using a microplate reader. The percentage inhibition of ABTS radical scavenging activity was calculated using the following equation:

(1) $$\%Inhibition=\frac{\left[({Abs}_{control}-{Abs}_{blank\;control})-({Abs}_{sample}-{Abs}_{blank\;sample})\right]}{\left({Abs}_{control}-{Abs}_{blank\;control}\right)}\times 100\%$$

The data are expressed as the mean ± standard deviation (SD) of % inhibition (triplicate, *n* = 3).

#### 4.2.2. DPPH• Scavenging Assay

According to a previous study, the protocol for DPPH radical scavenging assay is followed and slightly modified from Tadtong et al., 2025 [51].

A 0.1 mM DPPH radical solution was prepared by dissolving 2,2-diphenyl-1-picrylhydrazyl (DPPH) with absolute ethanol. The solution was mixed thoroughly, protected from light by covering the container with aluminum foil, and stored to prevent photo-induced oxidation. Honokiol and magnolol were diluted from the stock solution to obtain five different concentrations, varying from 20 to 100 µM.

For the antioxidant activity assay, 100 µL of honokiol, magnolol, and the mixture of honokiol (H) and magnolol (M) at molar ratios of H/M 1:3, 1:1, 3:1 solutions (at final concentration is 10–50 µM) was added to 100 µL of DPPH radical solution in individual wells of a 96-well microplate. The reaction mixture was incubated prior to measuring absorbance at 517 nm. The percentage inhibition of DPPH radical scavenging activity was calculated using Equation (1):

The data are expressed as the mean ± standard deviation (SD) of % inhibition (triplicate, *n* = 3).

#### 4.2.3. Ferric Reducing Antioxidant Power (FRAP) Assay

According to a previous study, the protocol for FRAP assay is followed and slightly modified from Tantinithiphong et al., 2025 [26].

The FRAP working reagent was freshly prepared by mixing 300 mM acetate buffer, 20 mM FeCl_3_·6H_2_O, and 10 mM TPTZ in a volumetric ratio of 10:1:1. The solution was thoroughly homogenized, sealed, and wrapped in aluminum foil to minimize light-induced oxidation. A series of five FeSO_4_·7H_2_O standard solutions at different concentrations were prepared to construct the calibration curve. Each test sample was diluted from its stock solution to a single working concentration for comparison with the Fe^2+^ standard curve.

For the assay, 40 µL of honokiol, magnolol, and the mixture of honokiol (H) and magnolol (M) at molar ratios of H/M 1:3, 1:1, 3:1 solution (at final concentration of honokiol, magnolol, and the mixture of honokiol (H) and magnolol (M) at molar ratios of H/M 1:3, 1:1, 3:1, and Trolox is 50 µM) was added to 160 µL of FRAP reagent in each well of a 96-well microplate. The reaction mixture was incubated in the dark for 30 min. Absorbance was then measured at 593 nm using a microplate reader.

The FRAP value was calculated using the linear regression equation derived from the FeSO_4_ standard curve, according to the following formula:

(2) $$\mathrm{FRAP}\mathrm{value}=\frac{\left({Abs}_{sample}-intercept\right)}{slope}$$

where:

- Abs_sample_ is the absorbance of the tested sample at 593 nm;
- Intercept is the y-intercept of the FeSO_4_ standard curve;
- Slope is the slope of the FeSO_4_ standard curve;
- The FRAP values were expressed as µM of ferrous (Fe^2+^) equivalent (triplicate, *n* = 3).

### 4.3. Computer Molecular Analysis

#### 4.3.1. Molecular Orbital Energy Analysis

According to a previous study, the protocols for molecular orbital energy analysis are followed and slightly modified from Tantinithiphong et al., 2025 [26].

The molecular structure of magnolol or honokiol was constructed using Avogadro 1.2.0. software, followed by automated geometry optimization (Auto Optimize) to obtain an initial minimized structure. A geometry optimization (OPT run) was subsequently performed at the second-order Møller–Plesset perturbation theory (MP2) level of theory with the def2-SVP basis set. The optimized Cartesian coordinates (xyz format) obtained from this calculation were then used to prepare an ORCA input file (.inp). Further optimization was conducted, employing the B3LYP functional in combination with the def2-SVP basis set.

**Figure 10:**
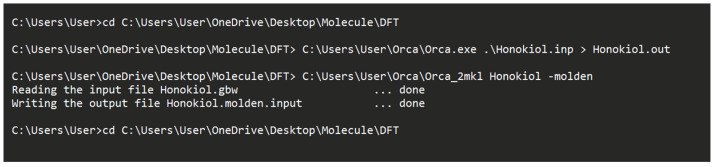
The ORCA calculation on an administrator command prompt with ORCA executed on these input files.

The ORCA calculation was executed using the prepared .inp file. The Command Prompt (cmd) was launched with administrator privileges, and the working directory was set to the folder containing the input file and executed to generate a Molden-format file as showed in Figure 10. The resulting .molden file was opened using IboView (v20211019) software to visualize the frontier molecular orbitals, specifically the highest occupied molecular orbital (HOMO) and the lowest unoccupied molecular orbital (LUMO).

#### 4.3.2. Drug-Likeness and ADME Analysis

According to a previous study, the protocols for drug-likeness and, Absorption, Distribution, Metabolism, and Excretion (ADME) analysis on SwissADME followed Ononamadu et al., 2021 [52].

The initial in silico evaluation of candidate compounds was conducted using the SwissADME web platform to assess their physicochemical characteristics and drug-likeness profiles. Drug-likeness was determined according to Lipinski’s rule of five and Veber’s criteria, with consideration of key molecular descriptors, including molecular weight, hydrogen bond donors and acceptors, lipophilicity (LogP), molar refractivity, total atom count, and topological polar surface area.

In addition, pharmacokinetic parameters were predicted using SwissADME (Swiss Institute of Bioinformatics; http://www.swissadme.ch/ (accessed on 10 January 2026). These predictions encompassed P-glycoprotein substrate specificity and blood–brain barrier (BBB) permeability to further evaluate the absorption and distribution potential of the selected compounds.

#### 4.3.3. In Silico Toxicity Analysis with ProTox 3.0

According to a previous study, the protocols for in silico toxicity analysis with ProTox 3.0 are followed from Banerjee et al., 2024 [34].

For in silico toxicity analysis, we used ProTox 3.0 (https://tox.charite.de/protox3 (accessed on 11 January 2026)). These predictions showed organ toxicity and metabolism of the selected compounds, such as hepatotoxicity, neurotoxicity, nephrotoxicity, respiratory toxicity, cardiotoxicity.

### 4.4. Cell Culture

According to a previous study, the protocol for cell culture is followed from Areebambud et al., 2023 [53].

SH-SY5Y cell line was maintained in 1:1 volume ratio of Eagle’s Minimum Essential Medium (EMEM): F-12 nutrient mixture, supplemented with 10% Fetal Bovine Serum (FBS) and 1% antibiotic–antimycotic solution at 37 °C in 5% CO_2_/90% air.

### 4.5. Neuronal Viability Assay of Honokiol and Magnolol

According to a previous study, the protocol for neuronal viability assay is followed and slightly modified from Areebambud et al., 2023 [53].

SH-SY5Y cells were treated with 0.001, 0.01, 0.1, 1, and 10 µM of honokiol and magnolol and the mixture of honokiol (H) and magnolol (M) at molar ratios of H/M 1:3, 1:1, 3:1 at 0.0001, 0.001, 0.01, 0.1, and 1 μM for 24 h, and the cell viability was measured via CCK-8 assay. CCK-8 solution was added to the treated cells and further incubated at 37 °C in 5% CO_2_/90% air for 2 h. After incubation, the absorbance value was read using a microplate reader at 450 nm.

### 4.6. Neuroprotective Assay

The assay was conducted on SH-SY5Y cells cultured in a 96-well plate and performed for three independent experiments.

#### 4.6.1. CCK-8 Assay

According to a previous study, the protocol for neuroprotective assay on CCK-8 assay is followed from Maicheen et al., 2026, and Samee et al., 2025 [54,55].

SH-SY5Y cells were seeded into 96-well plates at a density of 1 × 10^5^ cells/mL and further treated with the highest non-cytotoxic concentration of honokiol, magnolol (0.1 μM), and the mixture of honokiol (H) and magnolol (M) at molar ratios of H/M 1:3, 1:1, 3:1 (0.0001 μM) at 37 °C in 5% CO_2_/90% air in an incubator for 1 h, and then the cells were treated with Aβ_42_ with concentration 1.25 μM or MPP^+^ iodide with concentration at 1 mM (100 µL/well) for 24 h. Cell viability was assayed using CCK-8 method. The data were expressed as the mean ± standard deviation (SD) of % cell viability (triplicate, *n* = 3).

#### 4.6.2. Intracellular Reactive Oxygen Species (ROS) with Fluorescence Probe H_2_DCF-DA Assay

The protocols for detection of intracellular reactive oxygen species (ROS) with fluorescence probe H_2_DCF-DA assay are slightly modified from Areebambud et al., 2023 [53].

SH-SY5Y cells were seeded into 96-well plates at a density of 1 × 10^5^ cells/mL and treated with the designated neuroprotective compounds for honokiol, magnolol (0.1 μM), and the mixture of honokiol (H) and magnolol (M) at molar ratios of H/M 1:3, 1:1, 3:1 (0.0001 μM). At the end of the treatment period, the culture medium was aspirated and discarded. The cells were washed once with phosphate-buffered saline (PBS). Subsequently, 100 µL of 10 µM H_2_DCF-DA solution was added to each well, followed by incubation at 37 °C for 30 min.

After incubation, the H_2_DCF-DA solution was removed, and the cells were washed three times with PBS. Then, 100 µL of PBS was added to each well, and fluorescence intensity was measured using a microplate reader (excitation 485 nm, emission 528 nm).

Intracellular reactive oxygen species (ROS) levels were expressed as relative ROS percentage compared with the control group, calculated as follows:

(3) $$Relative\;ROS\;level\left(\%\;of\;control\right)=\left[\frac{{Fluorescence}_{sample}}{{Fluorescence}_{control}}\right]\times \;100\%$$

The data are expressed as the mean ± standard deviation (SD) of relative ROS level (% of control) (triplicate, *n* = 3).

### 4.7. Statistical Analysis

Statistical analysis of all experiments was performed using analysis of variance (one-way ANOVA), followed by Tukey’s HSD in GraphPad Prism 11.0.2. All data are expressed as mean ± standard deviation (SD) derived from three independent experimental replicates. Differences were considered significant only when the *p*-value was less than 0.05.

## 5. Conclusions

Oxidative stress plays a central role in the pathogenesis of Alzheimer’s and Parkinson’s diseases through the induction of mitochondrial dysfunction, lipid peroxidation, protein carbonylation, DNA damage and caspase-mediated apoptosis. Therefore, honokiol and magnolol are capable of reducing ROS accumulation and may provide significant neuroprotective benefits. Our study shows that honokiol may be a more potent antioxidant compound than magnolol due to its favorable electronic properties and stronger radical scavenging activity. Moreover, 0.1 μM honokiol, 0.1 μM magnolol, and H/M mixtures in all tested ratios (1:3, 1:1, 3:1) at 0.0001 μM revealed no significant differences in neuroprotective effects, and H/M mixtures in all tested ratios (1:3, 1:1, 3:1) at 0.0001 μM have synergistic effects for neuroprotective ability by reducing the amount of each individual compound required, which may help minimize concentration-related side effects while maintaining neuroprotective and antioxidant effects. One of the possible neuroprotective mechanisms of honokiol and magnolol is antioxidation; however, other possible mechanisms need to be proved. In addition, combined with favorable BBB permeability prediction, oral bioavailability, and predicted safety profiles, the honokiol–magnolol combination represents a promising candidate for mitigating the oxidative stress-associated mechanism and may be involved with other mechanisms of neurodegeneration in Alzheimer’s and Parkinson’s diseases.

## Figures and Tables

**Table 1:** Electronic parameters of honokiol and magnolol (eV).

Compounds	E_HOMO_	E_LUMO_	ΔE	First Ionization Potential (I)	Electron Affinity (A)	Electronic Potential (µ)	Chemical Hardness (η)	Electronegativity (χ)
Honokiol	−5.82	−0.36	5.46	5.82	0.36	−3.09	2.73	3.09
Magnolol	−5.76	−0.24	5.52	5.76	0.24	−3.00	2.76	3.00

Note: The results were interpreted comparatively, calculated with Koopmans’ approximation method from orbital energies from honokiol and magnolol.

**Table 2:** Physicochemical properties of honokiol and magnolol, computed by SwissADME.

No.	Compounds	MW (g/mol)	logP (WLogP)	TPSA (Å^2^)	HD	HA	RB	Lipinski’s Violations
Rules		<500	≤5	≤140	<5	<10		≤1
1	Honokiol	266.33	4.22	40.46	2	2	5	No
2	Magnolol	266.33	4.22	40.46	2	2	5	No

Note: MW—molecular weight; TPSA—Topological Polar Surface Area; HD—Hydrogen Donor; HA—Hydrogen Acceptor; RB—Rotatable bond.

**Table 3:** Pharmacokinetics parameters and bioavailability of honokiol and magnolol, computed by SwissADME.

Compounds	GI Absorption	BBB Permeant	P-gp Substrate	CYP 1A2 Inhibitor	CYP 2C19 Inhibitor	CYP 2C9 Inhibitor	CYP 2D6 Inhibitor	CYP 3A4 Inhibitor	Bioavailability Score
Honokiol	High	Yes	No	Yes	Yes	Yes	Yes	Yes	0.55
Magnolol	High	Yes	No	Yes	Yes	Yes	Yes	Yes	0.55

**Table 4:** Organ toxicity probability of honokiol and magnolol, computed by ProTox 3.0.

Compounds	Hepatotoxicity	Neurotoxicity	Nephrotoxicity	Respiratory Toxicity	Cardiotoxicity
Honokiol	Inactive (0.76)	Inactive (0.53)	Inactive (0.58)	Inactive (0.62)	Inactive (0.84)
Magnolol	Inactive (0.73)	Inactive (0.50)	Inactive (0.58)	Inactive (0.69)	Inactive (0.82)

Note: The data are shown as Active/Inactive (Probability data: xx.xx).

**Table 5:** Drug metabolism via the cytochrome P450 system of honokiol and magnolol, computed by ProTox 3.0.

Compounds	CYP1A2	CYP2C19	CYP2C9	CYP2D6	CYP3A4	CYP2E1
Honokiol	Inactive (0.63)	Active (0.81)	Active (0.88)	Inactive (0.78)	Inactive (0.70)	Inactive (0.99)
Magnolol	Inactive (0.57)	Active (0.82)	Active (0.87)	Inactive (0.79)	Inactive (0.64)	Inactive (0.99)

Note: The data are shown as Active/Inactive (Probability data: xx.xx).

## Data Availability

The original contributions presented in this study are included in the article. Further inquiries can be directed to the corresponding author.

## Abbreviations

The following abbreviations are used in this manuscript:

Aβ	Amyloid-beta
ABTS	2,2′-azino-bis(3-ethylbenzothiazoline-6-sulfonic acid)
ADME	Absorption, Distribution, Metabolism, Excretion
ADMET	Absorption, Distribution, Metabolism, Excretion, Toxicity
BBB	Blood–Brain Barrier
CCK-8	Cell counting kit-8
%cell viability	Percentage of cell viability
°C	Degree Celsius
CO_2_	Carbon dioxide
DNA	Deoxyribonucleic acid
DPPH	2,2-diphenyl-1-picrylhydrazyl
FRAP	Ferric Reducing Antioxidant Power
H/M	The mixtures of honokiol and magnolol in molar ratio
H_2_DCF-DA	2′,7′-dichlorodihydrofluorescein diacetate
HOMO	Highest Occupied Molecular Orbital
iROS	Intracellular Reactive Oxygen Species
LUMO	Lowest Unoccupied Molecular Orbital
MPP^+^	1-methyl-4-phenylpyridinium ion
MPTP	1-methyl-4-phenyl-1,2,3,6-tetrahydropyridine
MTT	3-(4,5-dimethylthiazol-2yl)-2,5-diphenyl tetrazolium bromide
μM	Micromolar
μL	Microliter
mg	Milligram
mL	Milliliter
